# Supplementary material for: Cerebrovascular claudin-5 isoform expression correlates with worsened stroke outcomes following thromboembolic stroke
Source: Fluids Barriers CNS. 2026 Apr 24;23:65. doi: 10.1186/s12987-026-00798-2 (PMC13126814; doi:10.1186/s12987-026-00798-2)
Supplement: Supplementary file 1 — Supplementary Material 1 [file 12987_2026_798_MOESM1_ESM.pdf]

Supplemental Data

Full Title: Cerebrovascular Claudin-5 Isoform Expression Correlates with Worsened Stroke Outcomes Following Thromboembolic Stroke

Authors: [1]Trevor S. Wendt Ph.D., [1]Henrik Andersson M.D., [1]Kajsa Arkelius Ph.D., [1]Saema Ansar Ph.D.

[1]Applied Neurovascular Research, Neurosurgery, Department of Clinical Sciences, Lund University, Klinikgatan 28, BMC C12, 222 42, Lund, Sweden

Short Title: Cerebrovascular Claudin-5 Isoform Following Stroke

Corresponding Author: Saema Ansar

Supplemental Figures

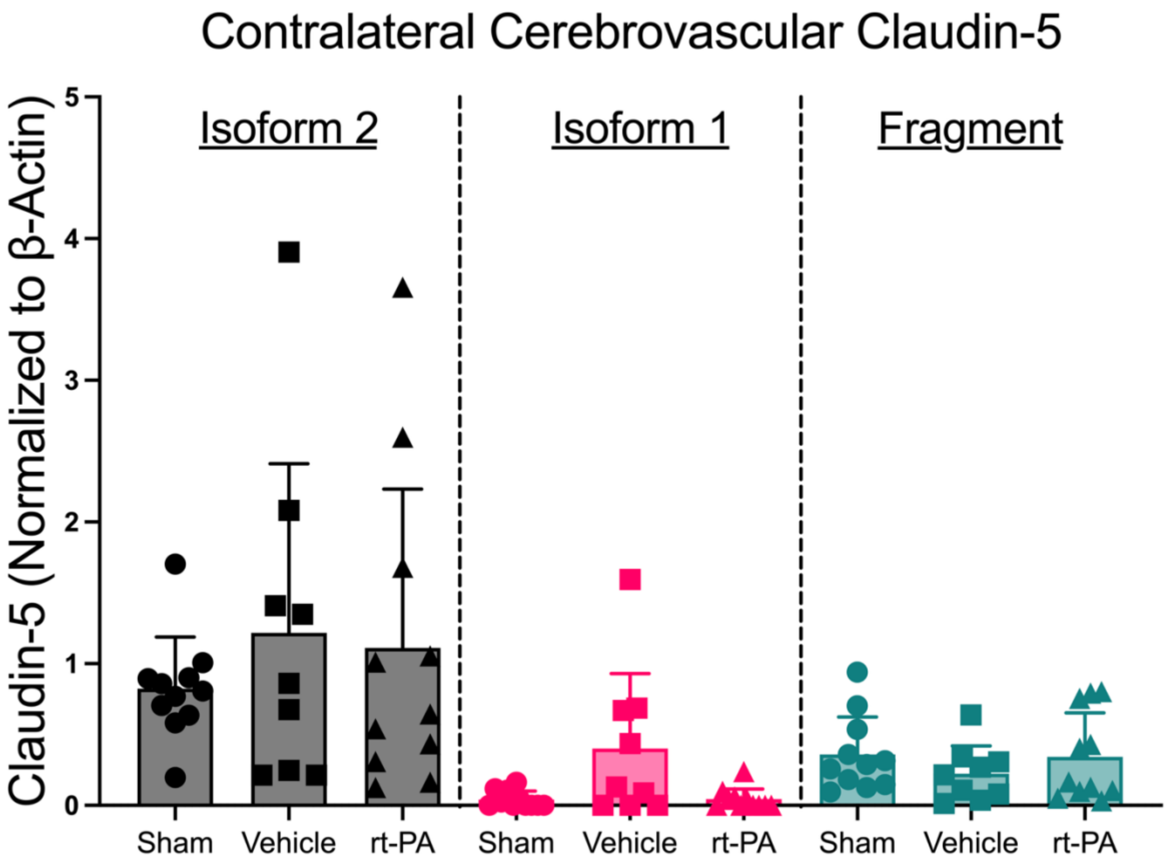

Supplemental Figure 1. Contralateral rt-PA Mediated Claudin-5 Protein Isoform Expression Following Thromboembolic Stroke. Densiometric quantification of claudin-5 isoforms normalized to corresponding β-actin in sham and at 24h post-stroke treated with either vehicle or delayed rt-PA in the isolated cerebrovasculature in the contralateral hemisphere represented as the relative densities of claudin-5/β-actin. N=9-11 individual animals. Non-parametric Kruskal-Wallis test was employed to assess differences between treatment groups.

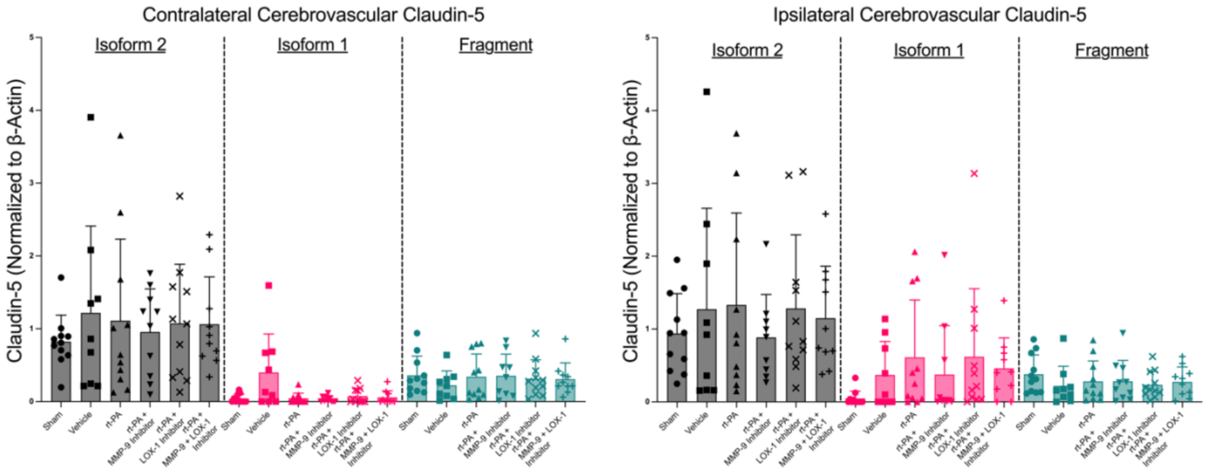

Supplemental Figure 2. Claudin-5 Protein Isoform Expression Following Thromboembolic Stroke and Experimental Treatments. Densiometric quantification of claudin-5 isoforms normalized to corresponding β-actin in sham and at 24h post-stroke treated with either vehicle or delayed rt-PA with the addition of either a selective MMP-9 inhibitor (JNJ0966), selective LOX-1 inhibitor (BI-0115), or the combination of both MMP-9 and LOX-1 inhibition in the isolated cerebrovasculature of the ipsilateral and contralateral hemispheres represented as the relative densities of claudin-5/β-actin. N=9-11 individual animals. Non-parametric Kruskal-Wallis test was employed to assess differences between treatment groups.

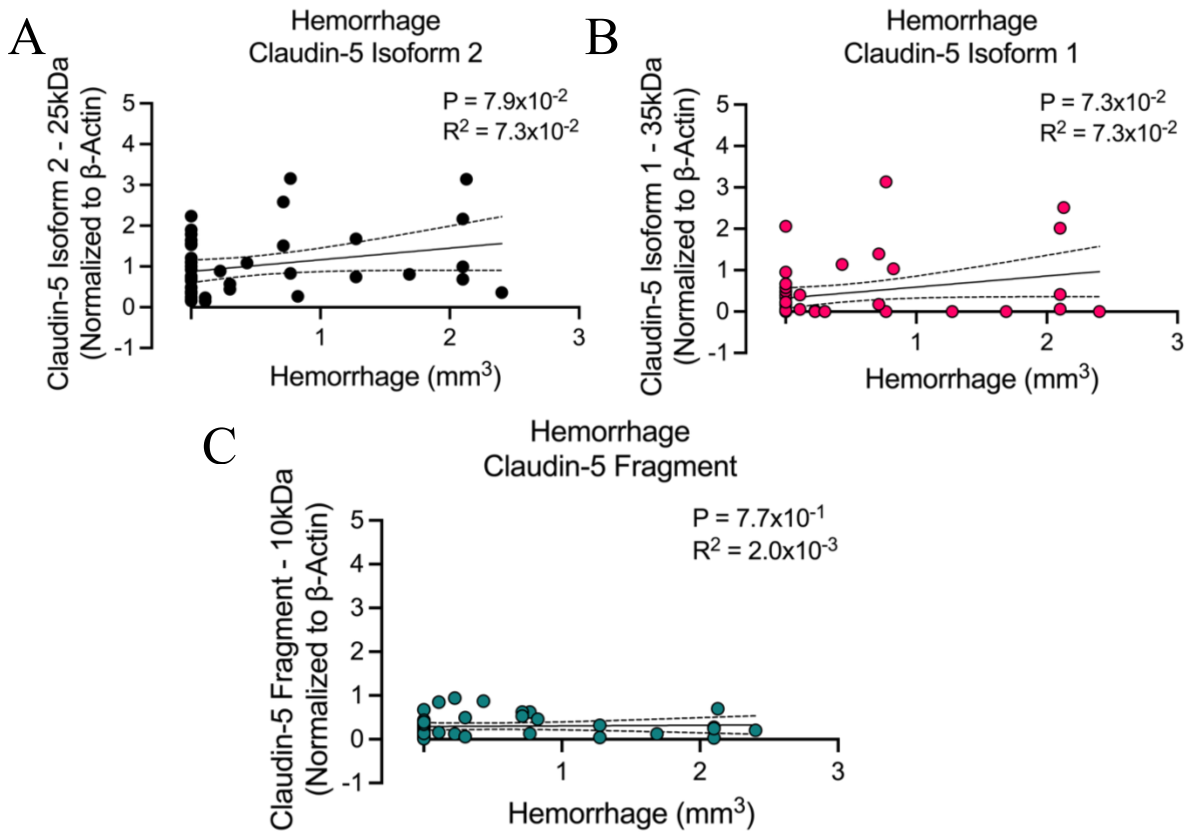

Supplemental Figure 3. Claudin-5 Protein Isoform Correlation to Non-Severe Hemorrhage Following Thromboembolic Stroke. Graphical illustration of the individual correlation between (**A**) claudin-5 isoform 2, (**B**) claudin-5 isoform 1, and (**C**) claudin-5 fragment and hemorrhage volume at 24h post-stroke onset. Simple linear regression was applied, and associated p-values (P) as well as R^2 values are shown within the graphical illustrations. N=43-45 individual animals.

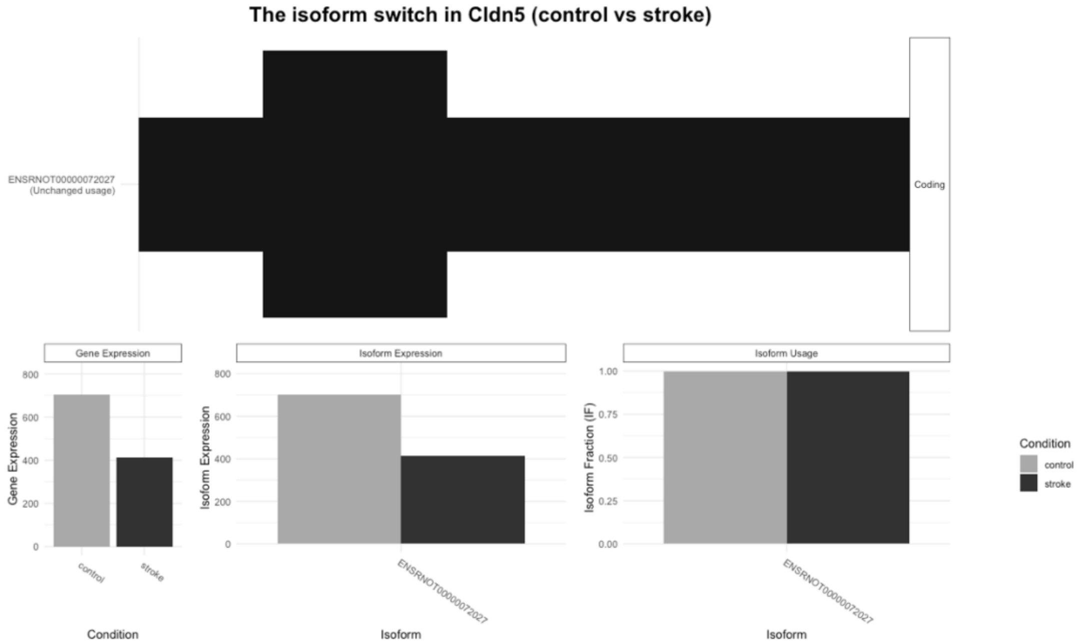

Supplemental Figure 4. Rattus Norvegicus IsoformSwitchAnalyzeR following tMCAO. IsoformSwitchAnalyzeR analysis of CLDN5 shows reduced total gene and isoform expression in tMCAO samples relative to controls (left and middle panels), while the relative usage fraction of the predominant transcript ENSRNOT00000072027 remains unchanged (right panel), indicating that the novel junction does not replace the primary annotated isoform.

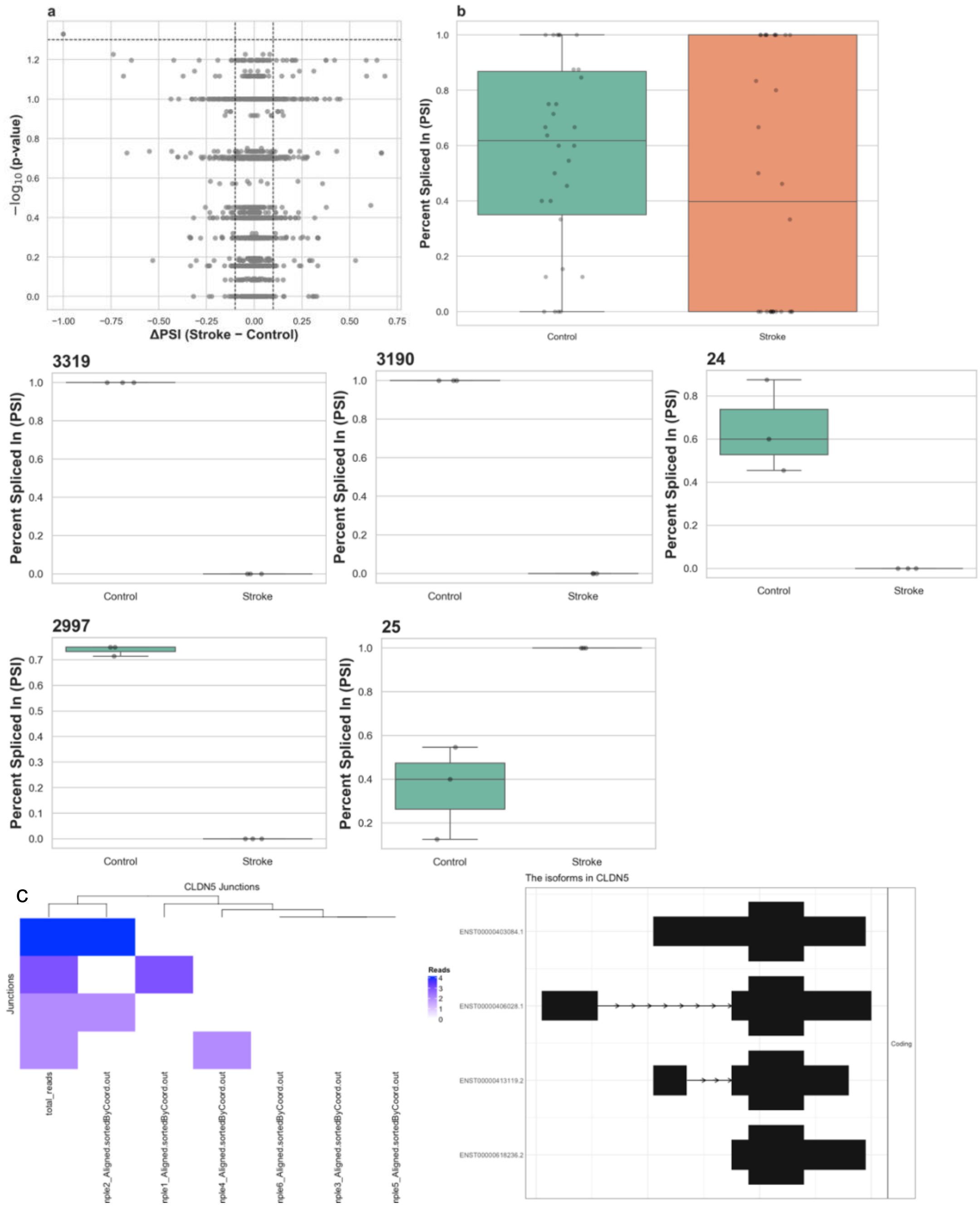

Supplemental Figure 5. Human brain endothelial splicing following ischemic-like injury. **(A)** Volcano plot showing ΔPSI (Stroke − Control) on the x-axis versus $-\log_{10}$(p-value) on the y-axis for all detected splicing events on human chromosome 22. Red points denote significant events (adjusted $p < 0.05$, $|\Delta PSI| > 0.1$), dashed lines indicate thresholds. (**B**) Distribution of Percent Spliced In (PSI) values for all events in control and stroke samples, with individual events overlaid. Right, PSI

distributions for the top five identified differentially spliced events. (**C**) Left, heatmap of *CLDN5*-specific junction read counts across all samples. Middle, predicted *CLDN5* isoform transcript structures. Right, boxplots of *CLDN5* isoform expression (TPM) showing markedly reduced expression in stroke relative to controls.

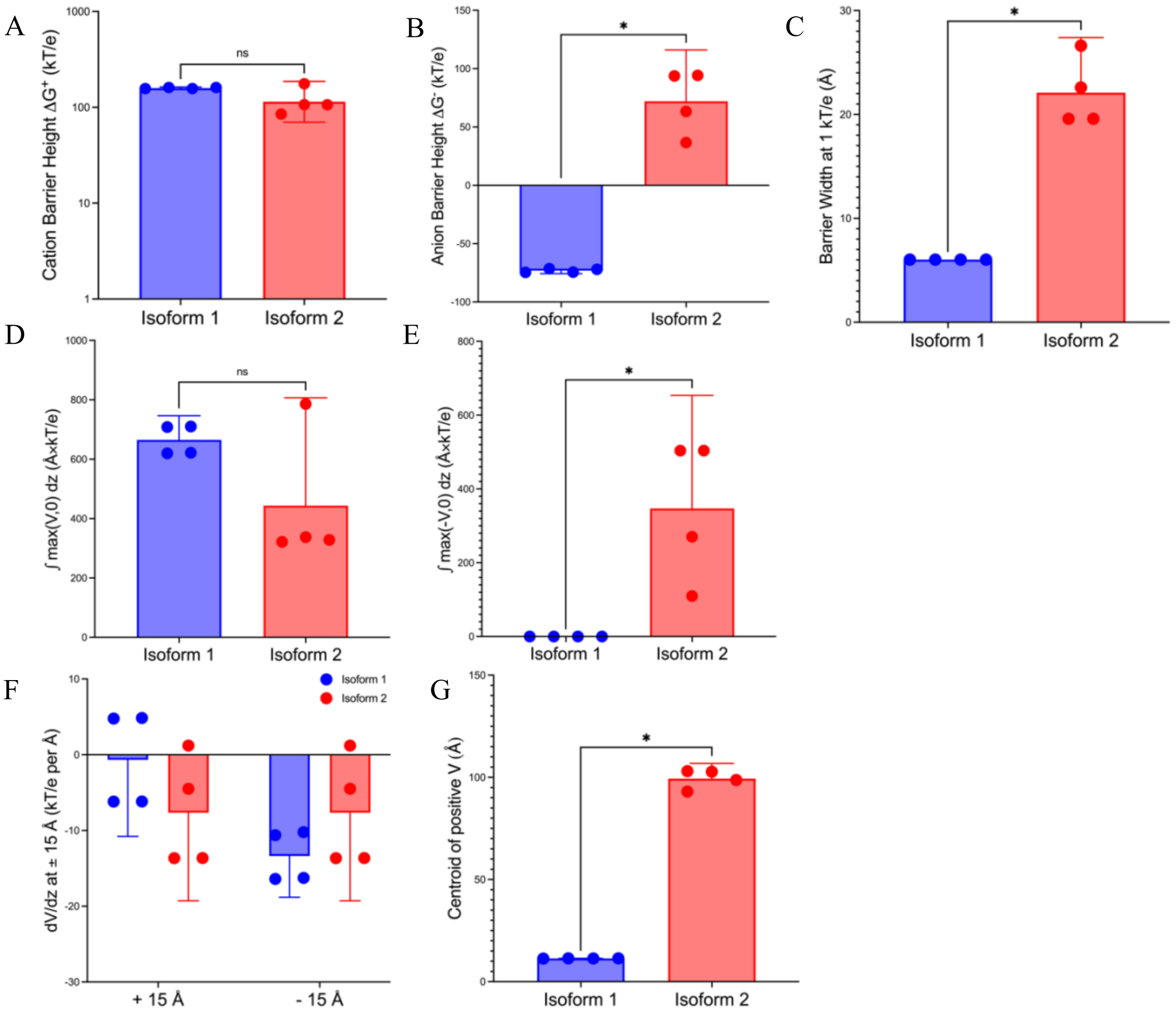

Figure 6. Isoform-specific claudin-5 paracellular permeability metrics. (A) Cation barrier height, $\Delta G^{+} = max_{z} V(z)$, (kT/e) within the core window [-15, +15] Å. **(B)** Anion barrier height, $\Delta G^{+} = max_{z}[-V(z)]$, (kT/e) within the same window. **(C)** Barrier width at 1 kT/e, axial width where $V(z) \geq 1$ kT/e (Å). **(D)** Positive area, $\int_{-15}^{+15} \max[V(z), 0]\, dz$ $(\text{Å} \times \frac{kT}{e})$, capturing barrier height × width relevant to cation exclusion. **(E)** Negative area: $\int_{-15}^{+15} \max[-V(z), 0]\, dz$ $(\text{Å} \times \frac{kT}{e})$, relevant to anion exclusion. **(F)** Mouth slopes, local field gradients $\frac{dV}{dz}$ $(\frac{kT}{e \times \text{Å}^{-1}})$ in $\pm$ 1 Å windows at $z = \pm 15$ Å (entrances). **(G)** Barrier centroid, centroid of the positive portion of $V(z)$ within [-15, +15] Å, $\bar{z} = \frac{\int z \max[V(z),0] dz}{\int \max(V(z),0] dz}$ (Å); positive values indicate extracellular displacement.

Electrostatic potentials were computed with APBS (310 K; values in kT/e; 1kT/e ≈ 26.7mV) and sampled along the CHARMM-GUI centerline $V(z)$. Points are individual seams (n=4 per isoform); graphs show mean ± 95% CI (bootstrap, 10,000 resamples). Group comparisons, where indicated, used two-tailed tests Mann–Whitney U; $P < 0.05$ considered significant. Visualization software includes VMD and PyMOL.

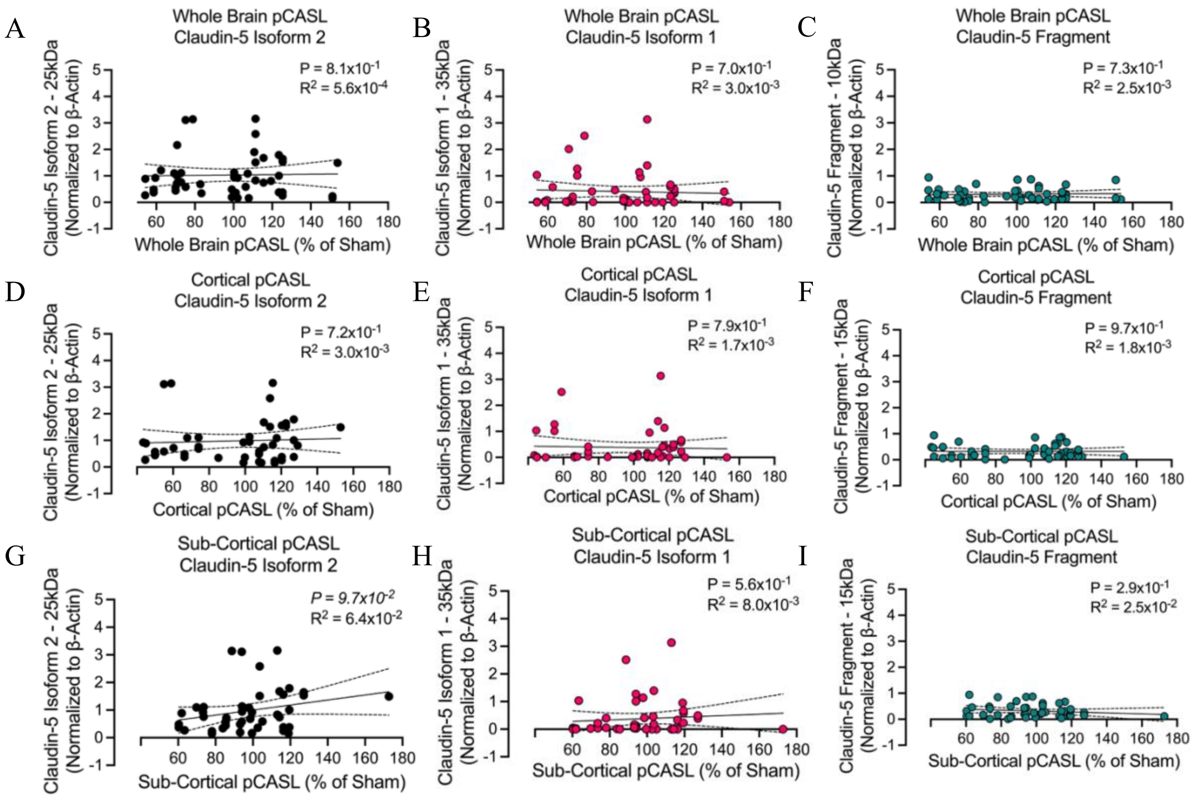

Supplemental Figure 7. Correlation of Ipsilateral Claudin-5 Isoform Expression with pCASL. (**A-C**) Graphical illustration of the individual correlation between (**A**) claudin-5 isoform 2, (**B**) claudin-5 isoform 1, and (**C**) claudin-5 fragment and whole brain pCASL at 24h post-stroke onset. Simple linear regression was applied, and associated p-values as well as R squared values are shown within the graphical illustrations. N=48-50 individual animals. (**D-F**) Graphical illustration of the individual correlation between (**D**) claudin-5 isoform 2, (**E**) claudin-5 isoform 1, and (**F**) claudin-5 fragment and cortical pCASL at 24h post-stroke onset. Simple linear regression was applied, and associated p-values as well as R squared values are shown within the graphical illustrations. N=44-46 individual animals. (**G-I**) Graphical illustration of the individual correlation between (**G**) claudin-5 isoform 2, (**H**) claudin-5 isoform 1, and (**I**) claudin-5 fragment and Sub-cortical pCASL at 24h post-stroke onset. Simple linear regression was applied, and associated p-values as well as R squared values are shown within the graphical illustrations. N=44-46 individual animals.
